# Enhancing ELISA Sensitivity: From Surface Engineering to Synthetic Biology

**DOI:** 10.3390/bios15070434

**Published:** 2025-07-06

**Authors:** Hye-Bin Jeon, Dong-Yeon Song, Yu Jin Park, Dong-Myung Kim

**Affiliations:** Department of Chemical Engineering and Applied Chemistry, Chungnam National University, Daejeon 34134, Republic of Korea; can9491@o.cnu.ac.kr (H.-B.J.); nicechris@cnu.ac.kr (D.-Y.S.); pppyj1113@o.cnu.ac.kr (Y.J.P.)

**Keywords:** enzyme-linked immunosorbent assay (ELISA), sensitivity, cell-free synthetic biology, in vitro diagnostic, biomarker

## Abstract

Accurate and sensitive detection of protein biomarkers is critical for advancing in vitro diagnostics (IVD), yet conventional enzyme-linked immunosorbent assays (ELISA) often fall short in terms of sensitivity compared to nucleic acid-based tests. Bridging this sensitivity gap is essential for improving diagnostic accuracy, particularly in diseases where protein levels better reflect disease progression than nucleic acid biomarkers. In this review, we present strategies developed to enhance the sensitivity of ELISA, structured according to the sequential steps of the assay workflow. Beginning with surface modifications, we then discuss the methodologies to improve mixing and washing efficiency, followed by a summary of recent advances in signal generation and amplification techniques. In particular, we highlight the emerging role of cell-free synthetic biology in augmenting ELISA sensitivity. Recent developments such as expression immunoassays, CRISPR-linked immunoassays (CLISA), and T7 RNA polymerase–linked immunosensing assays (TLISA) demonstrate how programmable nucleic acid and protein synthesis systems can be integrated into ELISA workflows to surpass the present sensitivity, affordability, and accessibility. By combining synthetic biology-driven amplification and signal generation mechanisms with traditional immunoassay formats, ELISA is poised to evolve into a highly modular and adaptable diagnostic platform, representing a significant step toward the next generation of highly sensitive and programmable immunoassays.

## 1. Introduction

The detection and quantification of low-abundance molecular biomarkers play a critical role in in vitro diagnostics (IVD). Clinical diagnostics routinely relies on diverse biomarkers, including nucleic acids, proteins, metabolites, and hormones, each providing complementary information about disease states [1,2,3]. Nucleic acid tests offer exceptional sensitivity for detecting pathogen DNA or RNA in infectious diseases and assessing genetic risk factors, while protein biomarkers provide direct, dynamic readouts of physiological states, critical for monitoring hormonal dysfunctions, cardiovascular diseases, and inflammatory disorders.

Advances in molecular diagnostics have improved the sensitivity and specificity of these approaches, supporting more precise and integrated disease management. However, relying solely on nucleic acid-based assays has limitations in capturing current disease activity, particularly in chronic or non-infectious diseases [4,5,6]. For example, in cancers, cardiovascular disorders, neurodegenerative diseases, and autoimmune conditions, nucleic acid biomarkers may indicate risk or predisposition rather than active disease progression [7,8,9,10]. Moreover, gene expression levels often fail to correspond to protein-level abnormalities [11,12,13]. This underscores the important role of measuring protein biomarkers to provide real-time insights into disease states and highlights the need for developing highly sensitive, specific, and accessible protein detection methods to support comprehensive diagnostic strategies.

Among the various protein detection platforms available, immunoassays stand out for their specificity and versatility in clinical settings. For example, immunoagglutination tests offer rapid, simple detection but limited sensitivity, while lateral flow immunoassays provide point-of-care convenience with moderate sensitivity levels [14,15]. Enzyme-linked immunosorbent assay (ELISA), however, remains the gold standard for quantitative protein biomarker detection due to its high sensitivity, robustness, accessibility, and adaptability [16,17,18]. Nonetheless, a significant sensitivity gap remains between ELISA and nucleic acid tests. While nucleic acid tests can achieve detection limits in the atto- to femtomolar range, conventional ELISA methods are limited to the pico- to nanomolar range, making them substantially less suitable for detecting low-abundance biomarkers. Bridging this sensitivity gap would greatly improve diagnostic accuracy, allowing for more precise disease monitoring by detecting both nucleic acids and proteins with comparable sensitivity.

A typical sandwich ELISA consists of three key steps: (1) immobilization of the capture antibody onto a solid surface with subsequent target biomarker binding; (2) binding of the detection antibody to the immobilized biomarker; and (3) signal generation and measurement via an enzyme label on the detection antibody [16]. Wash steps are introduced between each step to remove unbound molecules, thereby minimizing background noise and improving specificity. Unlike nucleic acids, which can be amplified using PCR or isothermal amplification techniques, proteins lack an intrinsic amplification mechanism. As a result, efforts to enhance ELISA sensitivity have primarily focused on optimizing capture antibody immobilization to improve biomarker retention (steps 1 and 2) and developing efficient signal amplification strategies to boost detection sensitivity (step 3).

**Figure 1:**
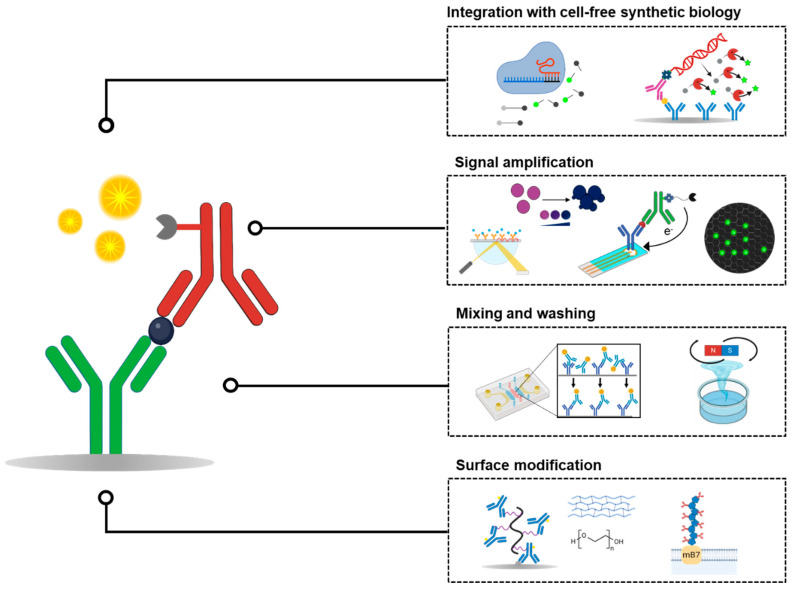
Layered approach to improving the sensitivity of ELISA. This review organizes the strategies for enhancing ELISA sensitivity according to their functional levels within the assay, starting from surface modification at the base, through mixing and washing improvements, signal amplification, and finally cell-free synthetic biology, building upward in a stepwise manner.

In this review article, we examine recent advancements aimed at enhancing ELISA sensitivity, first with a focus on improving biomarker capture efficiency and signal amplification techniques. We then explore the potential of cell-free synthetic biology as a novel approach to expand and enhance signal generation in ELISA (Figure 1). While numerous reviews have discussed optimization strategies for ELISA, including improved antibody immobilization and advanced signal detection chemistries, only a few have addressed the emerging role of synthetic biology in this context [19]. This review aims to fill that gap by presenting a stepwise overview of recent developments throughout the ELISA workflow, from surface engineering and reagent handling to novel approaches for signal amplification. In particular, we highlight the integration of cell-free synthetic biology as a programmable and modular platform that can enhance both the sensitivity and flexibility of immunoassays. This offers a perspective that has not been covered in earlier ELISA reviews. We believe that a comprehensive understanding of ELISA optimization strategies, especially through the integration of cell-free synthetic biology, will contribute to the development of next-generation protein detection technologies with higher sensitivity, adaptability, and versatility for in vitro diagnostics.

## 2. Surface Modification Strategies for Enhanced Antibody Coating

The sandwich ELISA procedure typically begins with the coating of capture antibodies onto a solid surface, a step that significantly influences target immobilization efficiency. Traditionally, capture antibody coating relies on passive adsorption onto polystyrene microplates via non-specific hydrophobic interactions. However, this method often results in random antibody orientation and partial denaturation, reducing the number of functionally active capture antibodies and ultimately that of surface-immobilized target biomarkers. In addition, adsorption of non-specific targets or detection antibodies onto exposed surfaces can generate false-positive signals [20].

### 2.1. Use of Blocking Agents to Reduce Non-Specific Binding

To mitigate this, microplates are usually pre-coated with blocking agents such as bovine serum albumin, skim milk, and casein [21]. These blocking agents occupy uncoated surface areas, thereby reducing non-specific binding and stabilizing antigen-antibody interactions to minimize false-positive and false-negative signals. The effectiveness of coating can differ significantly depending on the type of coating agents used, which affects the sensitivity and accuracy of the assay [22,23].

### 2.2. Nonfouling Surface Modifications Using Synthetic Polymers and Polysaccharides

**Figure 2:**
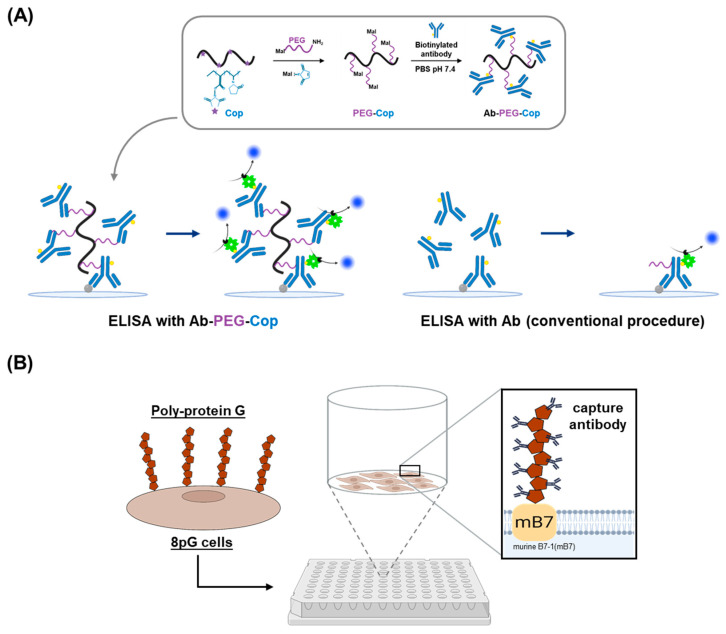
Surface modification strategies for enhancing capture antibody immobilization. (**A**) Schematic of multivalent antibody immobilization using antibody-conjugated polyethylene glycol-grafted copolymer, with a comparison against conventional ELISA employing free antibodies. Adapted from [24], with permission from the publisher. (**B**) Schematic of a microplate coated with cells expressing membrane-anchored poly-protein G to facilitate efficient capture antibody immobilization.

Nonfouling surface modifications using synthetic polymers offer an alternative strategy to prevent non-specific protein adsorption. For example, polyethylene glycol (PEG) can be used to confer resistance to non-specific protein interactions [24,25]. Recently, Trimaille et al. developed a PEG-grafted copolymer system (Figure 2A) that enables multivalent antibody conjugation, which not only minimizes nonspecific adsorption but also significantly enhances immunoassay sensitivity by improving the accessibility and avidity of antibodies towards target proteins [24]. Additionally, various polysaccharides such as dextran, chitosan, and hyaluronic acid have demonstrated successful reductions in non-specific adsorption [26,27]. For example, chitosan-coated surfaces have been explored as an alternative ELISA platform due to their ability to enhance protein immobilization while preventing non-specific interactions [28]. It was also demonstrated that polymer brushes can provide highly effective nonfouling surfaces, significantly improving the signal-to-noise ratio in ELISA assays [29].

### 2.3. Antibody Orientation Strategies for Enhanced Binding Efficiency

Along with minimizing non-specific binding, ensuring proper orientation of capture antibody substantially improves antigen accessibility, increases assay sensitivity, and enhances reproducibility. Several strategies have been developed to optimize antibody orientation and enhance detection efficiency. Protein A and Protein G immobilization are widely used methods for orienting antibodies in ELISA [30]. These bacterial proteins, derived from *Staphylococcus aureus* and *Streptococcus* species, respectively, bind to the Fc region of antibodies, ensuring a uniform and stable orientation. However, commercial Protein G-based microplates are costly due to the requirement for purification and immobilization. As an alternative, Chen et al. proposed an approach of coating surfaces with Protein G-expressing cells (Figure 2B) [31]. These engineered cells were fixed onto poly-D-lysine-coated microplates, thereby eliminating the need for purified Protein G and allowing direct immobilization of capture antibodies. This strategy enhanced antibody-coating capacity through uniform Fc-specific binding on a high-surface-area cellular substrate.

The biotin-streptavidin system offers another highly effective strategy for controlled antibody orientation. The exceptionally strong biotin-streptavidin interaction ensures that antibodies are immobilized in a uniform and functional state, reducing variability [32,33]. However, this method requires prior biotinylation of the capture antibody, which can sometimes affect antibody functionality and introduce additional preparation steps [34,35]. Covalent crosslinking provides a permanent and stable attachment of antibodies to the solid surface, preventing antibody loss during wash steps. Moreover, this approach can be combined with antifouling surface modifications, such as polymer coatings, to simultaneously enhance antibody orientation and prevent non-specific binding [36,37].

The selection of the optimal surface modification strategy depends on factors such as assay sensitivity requirements, cost considerations, and compatibility with different antibody types. By integrating nonfouling coatings with precise antibody orientation techniques, ELISA assays can achieve enhanced sensitivity, improved signal-to-noise ratios, and greater reproducibility, helping overcome major barriers in highly sensitive and reliable protein biomarker detection. Beyond these surface modifications, emerging alternative ELISA carriers deserve consideration for further enhancing assay performance. Magnetic beads offering improved washing efficiency and paper-based platforms enabling low-cost applications represent promising directions that complement traditional microplate-based approaches [38,39]. These diverse carrier options expand ELISA’s versatility across different application contexts while addressing specific technical and practical challenges in biomarker detection.

## 3. Mixing and Washing Efficiency in ELISA

In conventional ELISA procedures, the interaction between protein biomarkers and antibodies depends solely on passive diffusion within a static assay reaction mixture [14]. This diffusion-driven process requires long incubation times to ensure efficient target binding, particularly when working with low-abundance targets. In addition, the need for manual intervention during washing steps between binding and detection reactions has been a long-standing challenge of conventional ELISA. Since each step requires removal of unbound reagents, washing is crucial for minimizing background signals and ensuring assay specificity. However, conventional manual washing procedures introduce variability and increase assay time. While introducing convective mixing could solve this problem, ELISA is typically performed in small reaction volumes, making it difficult to achieve efficient mixing simply by shaking the plates. Although automated liquid handling systems can facilitate mixing through repeated aspiration and dispensing, these systems are costly and could be impractical in standard laboratory settings.

### 3.1. Implementing Microfluidic Systems

In recent years, microfluidic systems have emerged as a versatile approach to improving immunoassay performance, particularly in the context of ELISA. The miniaturization of fluidic architecture not only conserves reagents and sample volumes but also enables automation, portability, and integration with digital diagnostic platforms [40,41,42]. By incorporating microfluidic techniques, cumbersome ELISA steps, such as manual mixing and washing, can be replaced with automated fluid manipulation in miniaturized microchannels. Early microfluidic ELISA systems faced limitations due to their dependence on external pumps and peripheral connections to mobilize reagents. However, recent advancements have enabled autonomous reagent transport without the need for externally powered equipment, making microfluidic ELISA platforms a more practical option [43,44].

For example, the “Optimiser” ELISA system, where each well of a microplate is connected to a spiral microfluidic channel (200 × 200 µm) that serves as a reaction chamber, significantly reduces reagent consumption, requiring as little as 1 µL of reagent per well, while maintaining high assay sensitivity [45]. Additionally, washing steps are streamlined using a simple capillary-driven flushing mechanism, triggered by placing an absorbent pad at the microchannel outlet. This eliminates the need for manual washing, reducing assay variability and processing time. Furthermore, the increased surface-area-to-volume ratio in microfluidic channels enhances binding efficiency and detection sensitivity.

In another approach, Uddin et al. integrated on-chip disposable pumps and valves into a microfluidic ELISA device, which is fully compatible with commercial 96-well plates [46]. A roller bar system selectively pressurizes PDMS-based microfluidic valves, enabling precise loading and removal of solutions throughout the ELISA process. This design minimizes manual liquid handling, allowing sequential reagent delivery without external pumps or complex fluidic connections. More recently, Yafia et al. developed a programmable capillary flow device using 3D printing to perform complex reaction sequences [47]. This system relies on structurally programmed capillary paths to direct fluid flow, eliminating the need for active pumping mechanisms. Applying this platform to chip-based immunoassays, they were able to demonstrate a fully automated and portable ELISA workflow that enhances efficiency without sacrificing high analytical performance.

These advances highlight the growing feasibility of fully integrated, pump-free microfluidic ELISA systems that support complex fluid manipulation, automation, and portability. Continued progress in this area is expected to bridge the gap between centralized laboratory testing and decentralized, more accessible diagnostics.

### 3.2. Implementing Micro-Stirring Mechanisms

Another effective strategy for improving the performance of ELISA is to enhance the mixing of reagents within microwells. This is particularly relevant to plate-based ELISAs, where limited diffusion can hinder efficient antigen-antibody binding.

To address this issue, Wang et al. developed magnetically driven micro-stirrers for the rapid and efficient mixing of ELISA reagents [48]. Their system utilizes iron oxide (Fe_3_O_4_) magnetic particles, which are aligned into rod-like structures under an external magnetic field. These structures are subsequently fixed via silica coating, enabling them to function as micro-spinning stirrers upon exposure to a rotating magnetic field. In addition to accelerating reagent mixing, this approach streamlines the washing procedure by modifying the silica-coated stirrers to serve as capture antibody substrates. In their three-well system (reaction well, washing well, and detection well), magnetic rods coated with capture antibodies were moved between wells by modulating an external magnetic field. This design facilitated automated reagent transfer, eliminating manual washing steps and improving reproducibility.

From a practical standpoint, micro-stirring approaches are attractive because they offer performance gains without requiring complex surface modification or high-cost detection platforms. Moreover, many stirring components, such as permanent magnets or bubble generators, can be readily integrated into low-cost, disposable formats.

### 3.3. Applying Acoustic Waves

Surface acoustic waves (SAWs) represent a promising and unconventional strategy for improving ELISA reaction kinetics through remote and finely tunable agitation of reagents. Unlike mechanical stirring, which requires physical components within the reaction well, acoustic wave systems deliver vibrational energy through a transducer placed beneath the well plate.

In a recent study, Zhang et al. applied SAWs for the mixing of ELISA reagents [49]. Their system incorporates an acoustic wave generator positioned beneath a 96-well plate, producing Rayleigh SAWs of varying wavelengths. Across various tested conditions, SAW application markedly improved reagent mixing efficiency, enabling shortened assay times or enhanced sensitivity under standard incubation periods.

One of the key advantages of acoustic wave-based mixing is its compatibility with conventional ELISA plate formats. The SAW transducer can be externally applied to a standard 96-well plate without requiring any modifications to the plate or reagents, enabling easy integration without specialized consumables. In addition to significantly reducing incubation times, this method maintains or even enhances analytical sensitivity. When appropriately tuned, it delivers uniform energy across wells, offering a contact-free way to accelerate ELISA reactions.

Together, these strategies streamline ELISA workflows, reduce manual intervention, and enhance sensitivity, making the assay faster, more reproducible, and broadly accessible. Continued improvements in these areas will help further refine ELISA into a more efficient, cost-effective, and high-throughput diagnostic tool, reducing the sensitivity gap between nucleic acid and protein biomarker detection in IVD.

## 4. Enhancing ELISA Sensitivity Through Alternative Signal-Generation Mechanisms

The final step of ELISA involves signal generation and measurement using a signal-generating modality, typically enzymes conjugated to the detection antibody. Traditionally, enzyme-linked detection methods, such as horseradish peroxidase (HRP) catalyzing the oxidation of 3,3′,5,5′-tetramethylbenzidine (TMB), have been widely used for optical signal generation [50,51]. However, the sensitivity achievable by this format is often insufficient for detecting low-abundance biomarkers. To address this limitation, various alternative signal-generation strategies have been developed to enhance detection sensitivity. These strategies include enzyme-mimicking metal nanoparticles (Nanozyme ELISA), plasmonic nanoparticles (Plasmonic ELISA), redox-active molecules for direct electrochemical signal transduction (Electrochemical ELISA), Raman-active nanoparticles (SERS-ELISA), and miniaturized single-molecule detection platforms (Digital ELISA).

### 4.1. Nanozyme ELISA

Nanozyme ELISA utilizes nanozymes, nanomaterials with intrinsic enzyme-mimicking catalytic activity, as alternatives to traditional enzyme labels. Composed of metal or metal oxide nanoparticles, these nanozymes offer superior stability, resistance to harsh conditions, and tunable catalytic properties, enabling signal amplification and improved sensitivity [50,52]. Unlike conventional enzymes, nanozymes exhibit greater robustness, longer shelf life, and cost-effectiveness. They have been particularly effective in detecting low-abundance biomolecules in clinical diagnostics, environmental monitoring, and food safety testing [53,54,55]. Additionally, nanozymes provide diverse signal outputs, making them adaptable to various assay formats [54,56,57]. For example, Draz et al. developed an on-chip Zika virus (ZIKV) detection method using platinum nanoparticles (PtNPs) conjugated with a monoclonal antibody. Upon virus binding, PtNPs catalyzed the decomposition of hydrogen peroxide (H_2_O_2_), generating microbubbles detectable with a smartphone camera, demonstrating a simple, highly sensitive detection approach [57].

### 4.2. Plasmonic ELISA

**Figure 3:**
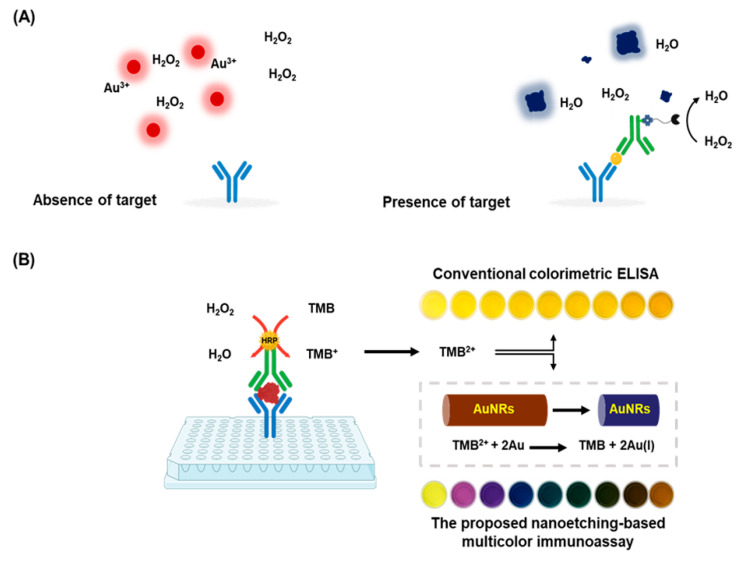
Enhanced colorimetric signal generation by plasmonic ELISA. (**A**) Schematic illustration of a plasmonic ELISA in which catalase-mediated decomposition of hydrogen peroxide modulates the formation and aggregation of gold nanoparticles, producing distinct colorimetric signals (red or blue). (**B**) Schematic of a nanoetching-based multicolor immunosensor for naked-eye detection, using TMB^2+^-mediated etching of gold nanorods. Adapted from [63], with permission from the publisher. (TMB: 3,3′,5,5′-Tetramethylbenzidine; AuNRs: gold nanorods).

Plasmonic ELISA employs the unique optical properties of gold or silver nanoparticles (AuNPs and AgNPs), nanostructures, or nanocomposites [58,59,60]. These materials exhibit strong localized surface plasmon resonance, enabling sensitive detection via absorbance changes. For example, the Stevens group developed a catalase-controlled colorimetric strategy based on H_2_O_2_-dependent AuNP growth to detect PSA and HIV-1 p24 at attomolar concentrations [61]. Catalase labeled to the detection antibody regulated H_2_O_2_ levels, affecting nanoparticle growth and color change (Figure 3A).

To improve visual readability, Guo et al. introduced a dual-color display system where AuNPs changed color based on analyte concentration [62]. Ma et al. further developed multicolor plasmonic immunoassays using enzyme-mediated etching and regrowth of noble metal nanoparticles, enabling semi-quantitative, naked-eye detection of biomarkers such as CEA, PSA, and H5N1 at low concentrations (Figure 3B) [63]. These strategies have been applied to detect small molecules, bacteria, and disease biomarkers [64,65]. In another example, Yang et al. developed a smartphone-based plasmonic ELISA platform utilizing gold nanorod etching to detect serum myoglobin, achieving a detection limit of 0.057 ng/mL [66].

### 4.3. Electrochemical ELISA

Electrochemical ELISA offers an alternative to colorimetric readouts by generating electrical signals through redox-active molecules. These systems immobilize antibodies on electrode surfaces, and after antigen-antibody binding, electrochemical transducers measure electrical changes such as conductance, current, potential, or impedance [67,68,69,70]. Owing to their high sensitivity, low cost, and resistance to color interference, E-ELISA platforms are promising for low-level biomarker detection. For example, Peng et al. developed an electrochemiluminescence (ECL)-based ELISA using gold nanoclusters (AuNCs) and MnO_2_-based signal quenching, achieving a TNF-α detection limit of 36 fg/mL—about two orders of magnitude lower than conventional ELISA [71]. Qin et al. developed a dual-wavelength ratiometric ECL immunosensor for amyloid-β_42_ detection, achieving attomolar-level sensitivity with excellent specificity [72]. Electrochemical ELISA is well suited for portable, automated, and point-of-care diagnostics, combining miniaturization with advanced materials and analytical techniques [73,74,75,76].

### 4.4. SERS-ELISA

Among the various strategies developed to improve ELISA sensitivity, the integration of surface-enhanced Raman scattering (SERS) provides a notable advancement in addressing the inherent limitations of absorbance- and fluorescence-based detection. In SERS-ELISA, metal nanoparticles functionalized with Raman reporter molecules (Raman tags) serve as detection probes [77,78]. Upon laser excitation, these Raman tags generate highly amplified signals via localized surface plasmon resonance (LSPR), enabling ultrasensitive, specific, and multiplex-capable quantification of target biomarkers [79,80]. Notably, SERS-ELISA excels at multiplexed detection, as the unique fingerprint spectra of Raman reporters allow simultaneous quantification of multiple biomarkers with high specificity and minimal signal overlap [81,82,83].

For instance, Lin et al. developed a nanogel-based SERS barcode system that enables logic-gated analysis of five matrix metalloproteinases using distinct metal–carbonyl Raman tags, achieving attomolar sensitivity and allowing accurate clinical staging of nasopharyngeal carcinoma based on multiplexed biomarker patterns [84]. This system is capable of detecting multiple cancer biomarkers with detection limits as low as 0.07 ng/mL, using only 5–10 µL of blood while maintaining high reproducibility. Similarly, Lee et al. established a rapid and cost-effective SERS platform for acute myocardial infarction screening, achieving detection limits of 6.56 fg/mL for creatine kinase muscle brain and 11.81 fg/mL for cardiac troponin I within 10 min, demonstrating its potential for emergency diagnostics and real-time monitoring [83].

### 4.5. Digital ELISA

**Figure 4:**
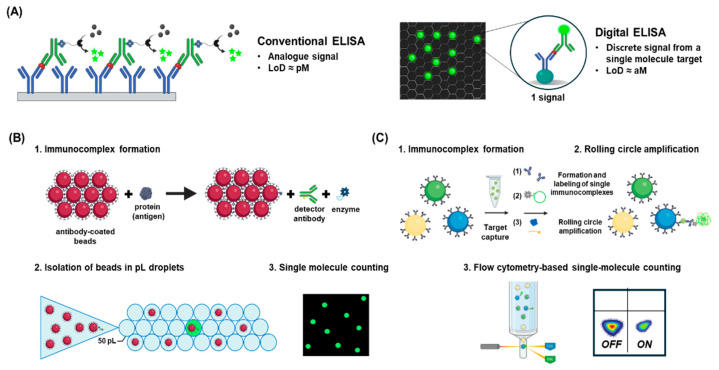
Advanced digital ELISA platforms for single-molecule target detection. (**A**) Conceptual comparison between conventional ELISA and digital ELISA platforms. (**B**) Workflow of droplet digital ELISA (ddELISA), involving immunocomplex formation, bead isolation in picoliter droplets, and digital signal counting. Adapted from [91], with permission from the publisher. (**C**) Schematic of the MOSAIC method, which uses bead-based rolling circle amplification and fluorescent probe hybridization for flow cytometry-based digital readout. Adapted from [92], with permission from the publisher.

In addition to advanced signal-generation methods, miniaturizing ELISA to the single-molecule level has enabled further sensitivity improvements. Rissin et al. introduced the single molecule array (Simoa) platform, which uses bead-based ELISA in femtoliter-sized microwells, allowing digital quantification of signal-positive beads across hundreds of thousands of wells [85,86,87]. In Simoa, target protein binding generates enzyme-mediated signals on beads, following the same principle as conventional ELISA (Figure 4A). This approach enhances sensitivity by confining reactions to small volumes and improving capture efficiency. For example, Kim et al. achieved a 2 aM detection limit for PSA, while Kan et al. improved it to 0.7 aM for IL-17A by optimizing bead distribution [88,89]. The Simoa HD-1 analyzer offers attomolar sensitivity and multiplexing, with over 1200-fold greater sensitivity than conventional ELISA [90].

Building on this, droplet digital ELISA (ddELISA) integrated digital ELISA with droplet microfluidics, using picoliter droplets as reaction chambers (Figure 4B). Cohen et al. demonstrated ddELISA with 25-fold higher sensitivity than Simoa, enabling quantification of LINE-1 ORF1p in serum [91]. Wu et al. further developed the MOSAIC platform, combining on-bead rolling circle amplification with flow cytometry-based detection (Figure 4C) [92]. This system enabled multiplexed quantification of up to eight analytes at attomolar levels in saliva and plasma. More recently, Zhang et al. introduced a barcoded version of MOSAIC that improved multiplexing accuracy while maintaining attomolar sensitivity, enabling five-protein detection from just 9 μL of blood [93].

Collectively, these platforms represent significant progress in immunoassay development, moving from analog measurements toward digital single-molecule resolution. By combining miniaturization, compartmentalization, and advanced signal amplification with multiplexed readouts, digital ELISA systems now achieve sensitivity comparable to nucleic acid diagnostics, expanding ELISA’s utility as a compact, automated, and versatile diagnostic tool [94,95,96].

## 5. Implementing Cell-Free Synthetic Biology to Improve the Sensitivity and Flexibility of ELISA

As discussed above, improving signal generation is key to enhancing ELISA sensitivity, approaching that obtainable with nucleic acid tests. While many strategies seek to replace traditional enzyme-based systems with alternative signal-generating methods, approaches that retain the familiar ELISA framework using standard reagents remain valuable for broad adoption.

Cell-free synthetic biology offers a programmable, in vitro platform that replicates cellular synthesis without the constraints of living systems [19,97,98,99,100]. Originally developed for protein expression, this technology is increasingly applied to bioassays, including ELISA, to produce functional biomolecules in response to target signals [101,102,103,104,105]. Since natural cells synthesize large quantities of proteins and nucleic acids under genetic control, translating this ability into a cell-free format enables new routes for signal amplification and assay customization [106,107,108].

Recent studies have integrated programmable synthetic components into the ELISA workflow, coupling antibody-based recognition with nucleic acid-driven amplification [105,109,110]. These hybrid approaches retain the standard assay format while overcoming its sensitivity limitations through in situ biosynthesis and signal enhancement.

### 5.1. Immuno-PCR

Immuno-PCR represents an early and influential example of incorporating nucleic acid amplification into ELISA to enhance detection sensitivity [111,112,113]. It relies on antibody–DNA conjugates, with the DNA serving as a template for PCR amplification, thereby allowing the detection of very low concentrations of antigens. While the advantage of signal amplification is clear, often improving detection limits by 100- to 10,000-fold, several limitations hinder its practical use [114,115]. Notably, immuno-PCR requires thermal cycling, making it dependent on specialized instrumentation and less suitable for point-of-care settings that depend on simple kits or microfluidic chips. Although isothermal amplification methods, such as rolling circle amplification or recombinase polymerase amplification, are being actively explored to address this issue, they currently face challenges such as high enzyme cost and relatively low amplification efficiency [110,116,117]. Furthermore, the need for technical expertise in both PCR and ELISA, combined with the complexity of multi-step protocols, presents a barrier to routine use. High background noise due to non-specific amplification and the difficulty of fully controlling hybridization and wash steps also impair test accuracy [118].

These limitations underscore the need for alternative approaches that retain the sensitivity benefits of nucleic acid amplification while offering simplified workflows and enhanced flexibility.

### 5.2. CRISPR-Linked Immunosorbent Assay (CLISA)

**Figure 5:**
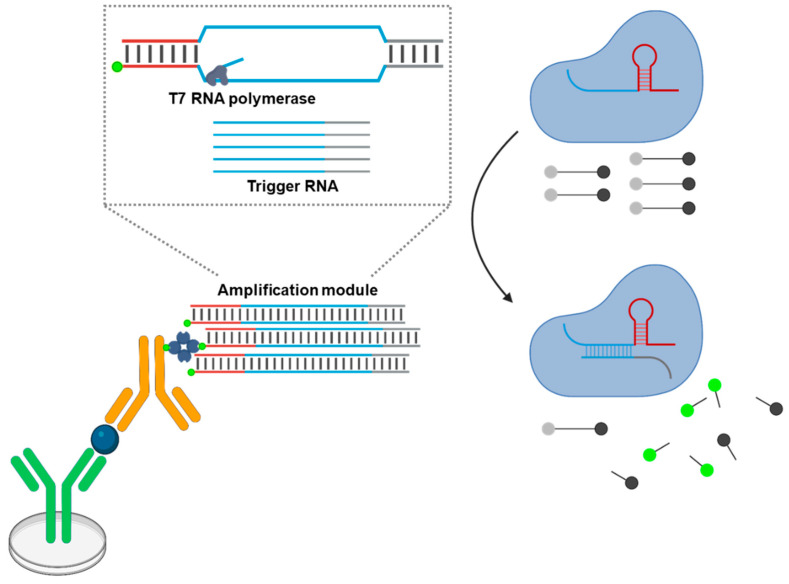
Schematic of CRISPR/Cas13a signal amplification linked immunosorbent assay for femtomolar protein detection. In a CLISA assay, the antigen is captured by a primary antibody and recognized by a biotinylated secondary antibody. A biotin-labeled DNA template is transcribed by T7 RNA polymerase, and the resulting RNA activates CRISPR/Cas13a collateral cleavage, enabling dual signal amplification proportional to antigen concentration. Adapted from [109], with permission from the publisher.

The CRISPR/Cas system has become a widely used tool in nucleic acid diagnostics, largely due to its trans-cleavage activity that enables rapid and high-turnover degradation of ssDNA or RNA [119,120,121]. A notable advancement is the integration of CRISPR/Cas into immunoassays, giving rise to the CRISPR-linked immunosorbent assay (CLISA) (Figure 5) [109]. In this strategy, enzyme tags in sandwich ELISA are replaced with a DNA sequence containing a T7 promoter. Upon antigen binding, T7 RNA polymerase transcribes this DNA into RNA, which is recognized by CRISPR-Cas13a, triggering the collateral cleavage of fluorophore-quencher, labeled reporter RNA. This enables highly sensitive detection of biomarkers such as IL-6 and VEGF, with detection limits of 2.29 fM and 0.81 fM, respectively.

To simplify the workflow, Li et al. developed the CRUISE platform, which uses biotin–ssDNA–antibody conjugates to directly activate Cas12a without requiring RNA transcription [122]. CRUISE supports flexible integration into ELISA formats and has demonstrated detection limits as low as 1 fg/mL (~50 aM). However, the reliance on streptavidin-based conjugation may cause steric hindrance, affecting binding efficiency.

Paialunga et al. addressed these issues by developing the CIA platform, which uses covalently linked Ab–DNA conjugates to activate Cas12a directly, eliminating enzymatic conversion steps [123]. This streamlined system, tested in both plate-based (CIA) and magnetic bead (CIMA) formats, enabled sensitive detection of SARS-CoV-2 spike proteins in both buffer and undiluted saliva, surpassing commercial ELISA kits in sensitivity and ease of use.

Despite their promise, CLISA and related methods face challenges, including labor-intensive conjugate preparation, non-specific background due to high sensitivity, and integration hurdles with standard immunoassay workflows. Overcoming these barriers is essential for broader adoption of CRISPR-enhanced immunoassays.

### 5.3. Expression Immunoassay

**Figure 6:**
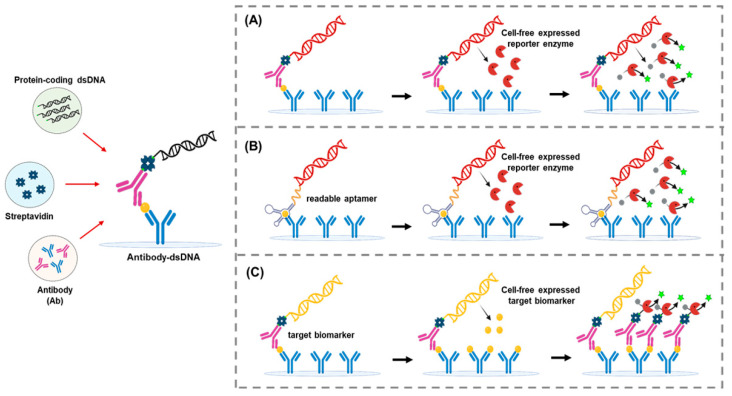
Advanced expression ELISA harnessing cell-free protein synthesis for biological signal amplification. (**A**) Schematic of enhanced immunoassay signal amplification using an *Escherichia coli* extract-based cell-free protein synthesis system. Adapted from [105], with permission from the publisher. (**B**) Schematic illustration of the aptamer-linked in vitro expression assay (ALIVE). Adapted from [126], with permission from the publisher. (**C**) Schematic of the nucleic acid-templated target amplification assay (NATA). Adapted from [127], with permission from the publisher.

Expression immunoassay, like immuno-PCR, links detection antibodies to DNA molecules [124]. Unlike immuno-PCR, where the labeled DNA serves as the template for PCR amplification, the DNA label in expression immunoassay directs cell-free synthesis of enzyme molecules for signal amplification (Figure 6A). While the original version of expression immunoassay employed rabbit reticulocyte lysate as the source of translational machinery, its sensitivity enhancement was limited due to insufficient translational activity. Byun et al. devised a highly efficient translation module for expression immunoassays using an *Escherichia coli* extract [105,125], allowing the detection of α-fetoprotein at 7 femtomolar concentrations. Baek et al. improved the convenience of this approach by replacing the detection antibody with an engineered aptamer [126]. In this method, named aptamer-linked in vitro expression assay (ALIVE), the aptamer sequence was connected to the expression cassette of an enzyme, eliminating the need for a separate step to conjugate antibody and DNA (Figure 6B). When tested for thrombin, the ALIVE method achieved a limit of detection as low as 2.7 aM.

More recently, the same group extended the concept of expression immunoassay for producing recombinant forms of target proteins during ELISA [127]. In this method, named nucleic acid-templated target amplification (NATA), the detection antibody was conjugated to the DNA encoding the target protein itself. This allowed the amplification of low-abundance targets during the ELISA step, and the amplified target was measured using the generic reagents such as HRP and TMB (Figure 6C). By simply introducing a cell-free amplification step for target proteins, this approach achieved markedly enhanced sensitivity without requiring any reagents beyond those used in conventional ELISA.

### 5.4. TLISA

The most recent example of combining cell-free synthetic biology with ELISA is likely the work of Styczinski’s group. They harnessed the mechanism of target-assisted activation of a set of split T7 RNA polymerase. In their modular system, called TLISA (T7 RNA polymerase–linked immunosensing assay), two fragments of split T7 RNA polymerase are each fused to nanobodies that recognize the same target antigen [128]. Upon target binding, the proximity of the nanobodies enables reassembly of the polymerase, which then initiates transcription of a reporter gene, producing a detectable output, such as a colorimetric change. This design enables intrinsic signal amplification via transcription and translation within a cell-free system, removing the need for traditional enzyme labels. TLISA demonstrated effective detection of clinically relevant proteins such as SARS-CoV-2 spike protein and transthyretin, providing visible readouts in under an hour and showing compatibility with complex sample matrices, including human saliva and serum.

## 6. Conclusions

Over the past decades, ELISA has evolved from a conventional enzyme-based assay into a versatile analytical platform, incorporating advances in biochemistry, synthetic biology, and nanotechnology to achieve sensitivities comparable to nucleic acid diagnostics. As shown in Table 1, many emerging ELISA strategies have demonstrated detection limits in the femto- to attomolar range, highlighting the remarkable analytical capabilities of recent developments. However, these advanced approaches often rely on sophisticated and costly instrumentation, which poses significant challenges in terms of accessibility, scalability, and integration with point-of-care testing. This suggests that, despite remarkable technical progress, practical limitations must be addressed before widespread implementation can be realized. It should also be noted that while our discussion has primarily focused on sandwich ELISA formats for protein biomarkers, competitive ELISA remains essential for small molecules that cannot bind two antibodies simultaneously. Future strategies to improve sensitivity should therefore consider adaptations appropriate for different types of targets in addition to the protein biomarkers discussed herein.

Looking ahead, the convergence of emerging technologies provides a promising direction. By combining synthetic biology–based signal amplification, modular assay designs, and emerging molecular tools, these innovations are gradually overcoming the inherent limitations of traditional ELISA. Approaches that integrate synthetic biology-based signal amplification, modular assay design, and molecular engineering are gradually overcoming the limitations of traditional immunoassays. In particular, combining synthetic biology with diagnostic innovation is enabling programmable and reconfigurable assay formats that move beyond the constraints of classical enzyme kinetics and antibody–antigen interactions.

Rather than replacing the established ELISA framework, cell-free synthetic biology builds upon it by introducing flexible and responsive signal generation mechanisms. Combined with advances in nucleic acid engineering, CRISPR-based amplification, and innovative signal strategies, ELISA is being transformed from a static assay into a highly adaptive, customizable molecular platform. Ultimately, this shift marks a transition from analytical tools to adaptive diagnostic devices, pointing toward a future where immunoassays are defined not by their constraints but by their precision, programmability, and accessibility.

## Figures and Tables

**Table 1:** Comparative summary of recent ELISA innovations based on analytical performance and practical implementation factors.

Method	Analytes	Limit of Detection	Time to Result	Scalability	Cost	Reference
Nanozyme ELISA	HBV, HCV, ZIKV	250 copies/mL	50 min	High	Medium	[57]
Plasmonic ELISA	PSA, HIV-1 capsid antigen p24	10^−6^ ng/mL	7 h	Medium	Low	[61]
	Serum myoglobin	0.057 ng/mL	4 h	High	Medium	[66]
Electrochemical ELISA	Aβ_42_	2.6 fg/mL	3 h	Medium	Medium	[72]
Digital ELISA	≥20 cytokines and neuro markers (e.g., IL-6, Tau, Aβ42)	1~10 fg/mL	30 min–2.5 h	High	High	[90]
	IFN-γ, IL-2	0.51 fg/mL, 1.0 fg/mL	2–3 h	High	Medium	[91]
	IL-6, IL-10, IFN-γ, etc. (up to 8-plex)	0.3~4.8 pg/mL	3 h	High	High	[92]
CLISA	IL-6, VEGF	45.8 fg/mL, 32.3 fg/mL	4 h	Medium	Medium	[109]
	IFN-γ, EGFR	1 fg/mL	3–4 h	High	Medium	[122]
Expression ELISA	thrombin	0.1 pg/mL	4 h	Medium	Medium	[126]
	AFP, IL-6	0.414 pg/mL, 0.0126 pg/mL	4 h	High	Medium	[127]
TLISA	SARS-CoV-2 spike (S)	21 μg/mL	<1 h	Medium	Medium	[128]
